# Isolated Femoral Muscle and Bone Metastases Rarely Encountered in Testicular Seminoma

**DOI:** 10.1155/2013/780493

**Published:** 2013-05-12

**Authors:** Serkan Degirmencioglu, Burcu Degirmencioglu

**Affiliations:** ^1^Şehit Albay Karaoğlanoğlu, Str. Zafer Gökşin, Oncology Center, 20100 Denizli, Turkey; ^2^Nuclear Medicine Department of Denizli State Hospital, Denizli, Turkey

## Abstract

Isolated muscle and bone metastases are rarely encountered in patients with testicular seminomas. In the present study, a patient who was admitted with pain, loss of motion, and swelling in the right leg 20 months following surgery for stage I seminoma was presented. Hypermetabolic lesion was detected in the right femoral muscle and bone via positron emission tomography. After the presence of metastasis from seminoma was confirmed by biopsy, bleomycin, cisplatin, and etoposide, combination chemotherapy was administered to the patient.

## 1. Introduction

Seminomas account for more than half of testicular tumors. Radiotherapy after orchiectomy and radical lymph node resection is the basis for the treatment in stage I seminomas [1]. Relapses presenting as isolated muscle and bone metastases have been rarely reported in the literature. Bone metastasis in patients with seminomas frequently presents with thoracolumbar spine involvement. Fluoro-18 (F-18) fluorodeoxyglucose (FDG) positron emission tomography (PET) usually detects residual tumor after chemotherapy [2, 3]. FDG-PET is also recommended for initial staging. F-18 FDG uptake is higher in seminomas compared to nonseminomatous testicular tumors [2]. Herein we report a patient with isolated muscle and bone metastases in the right femur on a PET-computed tomography (CT) scan 20 months after therapy. Following confirmation of the metastasis via biopsy, chemotherapy was administered.

## 2. Case Report

**Figure 1:**
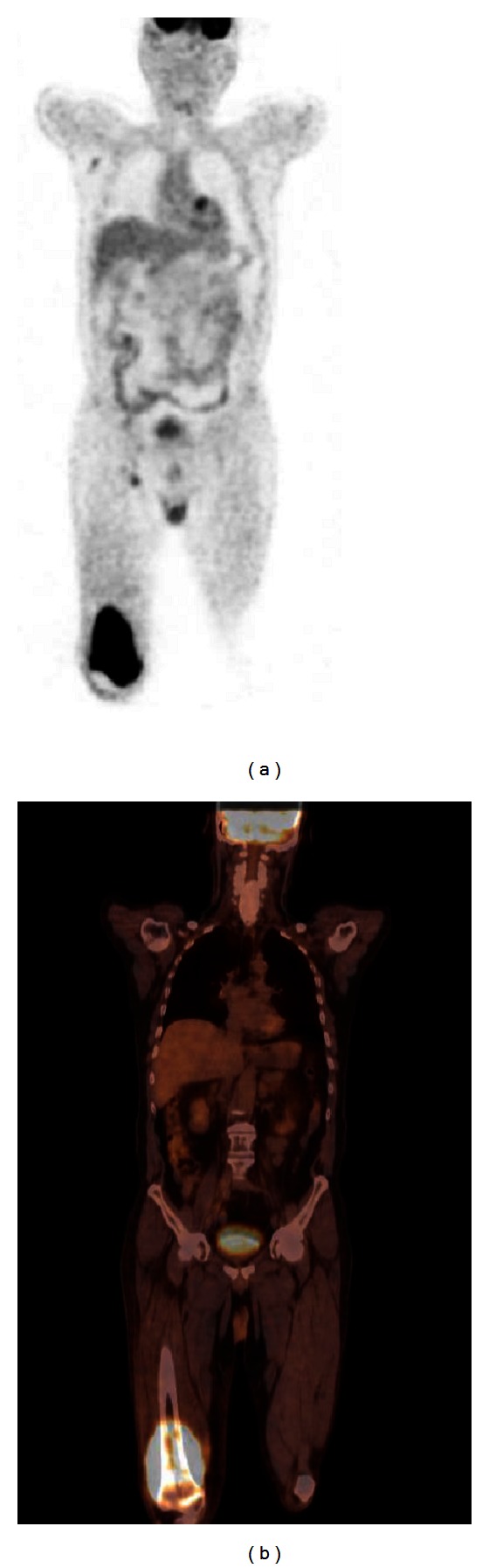
Hypermetabolic lesion, which begins from the middle part of the right femur and invaded the muscular tissue, as well as the bone tissue and bone marrow in the distal diaphysis of the femur, is observed on the PET (a) and fusion (b) images of the patient (SUVmax = 16.1).

**Figure 2:**
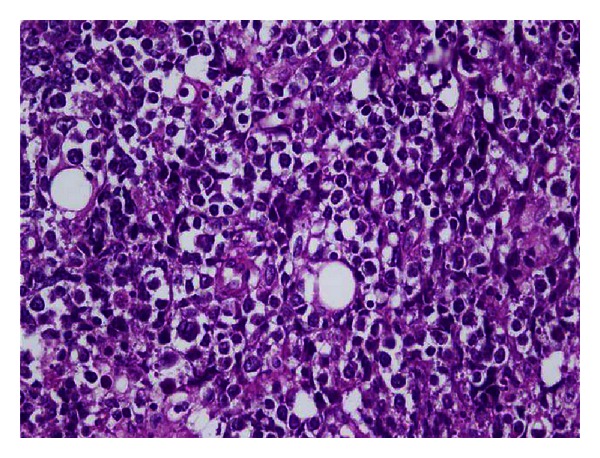
Seminoma infiltration that shows solid growth pattern (×40, H&E).

A 62-year-old male patient underwent biopsy 20 months ago due to swelling in his right testicle. He was diagnosed with a seminoma and underwent right orchiectomy and radical lymph node resection. The pathologic examination of the surgical specimen revealed a stage I seminoma. After receiving radiotherapy (24 Gy) to the para-aortic region, the patient declined chemotherapy. After 20 months of surgery, the patient, who was out of follow-up, was admitted to our polyclinic with pain, loss of motion, and swelling in his right leg. On physical examination, a 3.5 × 4 cm mass was detected in the distal part of the right femur beginning from the subcutaneous region and fixed to the bone. A magnetic resonance imaging (MRI) of the right femur revealed a loss in the normal bone signal in the distal metaphyseal-diaphyseal region of the femur, and the soft tissues showed uptake of contrast media around the bone in the postcontrast series, reaching up to 3.5 cm at the widest portion. The lactate dehydrogenase level of the patient was 479 IU/L (normal range, 125–243 IU/L), whereas the alpha-fetoprotein (AFP) and beta human chorionic gonadotrophin (HCG) levels, hemogram, and other biochemical analyses were within normal ranges. A PET-CT was performed to eliminate other likely metastases prior to surgery. The PET-CT of the patient revealed a hypermetabolic lesion beginning from the medial part of the right femur and extending to the tibial margin. The lesion extended over the intermuscular tissue in this region and invaded the muscular tissue, as well as the bone tissue, and bone marrow in the distal diaphysis of the femur (Maximum Standardized Uptake Value [SUVmax] = 16.1; Figure 1). Thereafter, the patient was transferred to the orthopedics department, where he underwent a biopsy. Soft tissue and bone biopsies were consistent with metastasis from seminoma (Figure 2). Bleomycin, cisplatin, and etoposide combination chemotherapy was planned.

## 3. Discussion

Testicular tumors are the most commonly encountered solid tumors in males between 15 and 35 years of age. The second peak in incidence occurs after the age of 60 years. Seminomas account for more than half of testicular tumors. The patients are most commonly admitted with a painless testicular mass [1]. The tumor is limited to the testicles in 80% of the patients at the time of diagnosis (stage I). A therapeutic response up to 99% is achieved with adjuvant radiotherapy after orchiectomy. Of stage I patients, the most common sites of relapse are the para-aortic lymph nodes and lungs [4]. Isolated muscle and bone metastases are rarely observed in pure seminomas [5, 6]. Generally, they present together with multiple visceral metastases.

In seminomas, FDG-PET plays an important role in the evaluation of residual masses after chemotherapy. In their series, Cremerius et al. [3] determined the sensitivity of PET to be 90% in the detection of residual tumor in patients with seminomas. Detection of residual tumors is important, since therapy protocols for these patients are different. In a study conducted by De Santis et al. [7] on 23 patients with seminomas, the sensitivity, specificity, positive predictive value (PPV), and negative predictive value (NPV) of FDG-PET were reported to be 89%, 100%, 100%, and 97%, respectively, in the detection of residual masses >1 cm in size. These values increase for the masses >3 cm in size [7].

Hain et al. [8] reported a specificity of 100% and a PPV of 100% via PET in the diagnostic staging of 31 patients with either seminomatous or nonseminomatous testicular tumors. The use of FDG PET-CT is beneficial in the selection of patients for follow-up to assess occult spread after orchiectomy in stage I seminomas. In this way, the patients at high risk for relapse can also be detected [2, 9]. The most appropriate therapy protocol for the patient is selected and the patient is protected from unnecessary therapy. While surgery is avoided in residual lesions >3 cm in size with negative FDG-PET findings, resection should be performed in lesions with positive FDG-PET findings, if possible [10].

Following surgery, radiotherapy is applied to the retroperitoneal area, particularly in stage I-II seminomas. Distant metastases were detected in 20% of such patients [2, 3], as in the present case. Relapses occur in 30% of patients within 2 years after orchiectomy [11]. The most common sites of metastases are the liver and lungs. More rarely, brain and bone metastases can also occur [12]. Cremerius et al. [3] reported a metastasis rate of 87% in 50 testicular tumors via PET; however, PET eliminated the metastases by 94% compared with CT. FDG-PET is superior to CT in the detection of metastases [13]. Testicular tumors spread through lymph nodes. In particular, the para-aortic chain, supradiaphragmatic nodes, and mediastinal and supraclavicular nodes are involved [12]. The lymphadenopathy that is detected by CT may consist only of reactive cells. The false positivity rate of CT in such patients is greater than 40% [14]. Therefore, PET-CT was preferred in the present case.

The number of cases diagnosed with stage I seminoma and relapsed solely with muscle and bone metastases, as in the present case, is limited in the literature [5, 6]. Only orchiectomy was applied in a case and followed up; MRI, which was performed after 22 months because of difficulty in walking, revealed spinal cord compression due to a pathologic fracture involving the 12th thoracic vertebra [5]. Orchiectomy and radiotherapy were applied in another case; MRI, which was performed after 24 months because of back pain, revealed a mass involving the 10th rib [6].

In their retrospective study on 650 cases with testicular tumors, Husband and Bellamy [12] defined unusual metastases, including 6 renal, 4 adrenal gland, 4 inferior vena cava, 3 muscle, 2 splenic, 1 gastric, 1 pelvic, 1 seminal vesicle, 1 prostate, and 1 pericardial [12]. In addition, diffuse splenic metastasis was detected after 4 years via FDG-PET in a patient who had been diagnosed with stage III seminoma, and a soft tissue metastasis that led to brachial plexus neuropathy in the right upper arm of a patient was detected 6 years after orchiectomy due to a seminoma [15].

## 4. Conclusion

In summary, after 20 months of surgery due to a diagnosis of seminoma, the patient, who was out of follow-up, presented to our policlinic with pain in the right leg and a right femoral mass was detected on physical examination. An isolated solitary metastasis was revealed via PET-CT, performed in order to discriminate other possible metastases. After the diagnosis was confirmed by biopsy, the patient was scheduled to receive chemotherapy.
